# An Autopsy Case of Reversible Cerebral Vasoconstriction Syndrome After a Severe Acute Respiratory Syndrome Coronavirus 2 Vaccination

**DOI:** 10.7759/cureus.59311

**Published:** 2024-04-29

**Authors:** Mai Shimura, Hirohisa Fujikawa, Masanobu Yazawa, Yuki Matsumoto, Mitsunori Yamada

**Affiliations:** 1 Department of Medical Oncology, National Cancer Center Hospital East, Chiba, JPN; 2 Department of Internal Medicine, Fujimi-Kogen Hospital, Fujimi-Kogen Medical Center, Nagano, JPN; 3 Center for General Medicine Education, School of Medicine, Keio University, Tokyo, JPN; 4 Department of Internal Medicine, Suwa Central Hospital, Nagano, JPN; 5 Department of Pathology, Shinshu University School of Medicine, Nagano, JPN; 6 Department of Brain Disease Research, Shinshu University School of Medicine, Nagano, JPN

**Keywords:** epilepsy, headache, autopsy, rcvs, covid-19, sars-cov-2 vaccination

## Abstract

A 73-year-old man with chronic obstructive pulmonary disease received the severe acute respiratory syndrome coronavirus 2 (SARS-CoV-2) mRNA vaccine. The following day, the patient developed a headache, followed by a tonic-clonic seizure and decreased consciousness. Magnetic resonance imaging of the head revealed no signs of stroke but multiple vasoconstrictions. Despite antiepileptic therapy, the seizure persisted, and the patient died 40 hours after vaccination. An autopsy revealed multiple brain ischemia without any vascular lesions, suggesting reversible cerebral vasoconstriction syndrome (RCVS). In this case, RCVS was diagnosed radiographically and pathologically. Our case suggests that RCVS could be a cause of headache and epilepsy following the SARS-CoV-2 mRNA vaccination.

## Introduction

The coronavirus disease 2019 (COVID-19) has become a threat to people and has spread globally. COVID-19 mRNA vaccines have been used worldwide for prevention [1]. Although it has been highly effective in preventing the disease, the vaccine can cause adverse effects such as headaches, fever, fatigue, and local reactions. Headache is the most common neurological side effect experienced by more than half of the recipients [1]. The causes of headaches range from tension-type headaches to strokes. Reversible cerebral vasoconstriction syndrome (RCVS) may occur after mRNA vaccination against the severe acute respiratory syndrome coronavirus 2 (SARS-CoV-2), causing severe headaches and death. There are several case reports of RCVS after mRNA vaccination; however, its pathophysiology is poorly understood. Herein, we report an autopsy case of RCVS after the third SARS-CoV-2 vaccination. This condition is rare in clinical practice; therefore, it needs to be reported to aid clinicians' decision-making regarding diagnosis and treatment.

## Case presentation

We present the case of a 73-year-old man hospitalized for acute exacerbations of chronic obstructive pulmonary disease. His past medical history includes hypertension, osteoporosis, and giant cell arteritis. The patient denied any past medical history of neurological disorders. The patient was administered salbutamol, ceftriaxone, or prednisolone. On the 38th day of hospitalization, the patient developed hospital-acquired pneumonia and was treated with the appropriate antibiotics (piperacillin and tazobactam).

**Figure 1:**
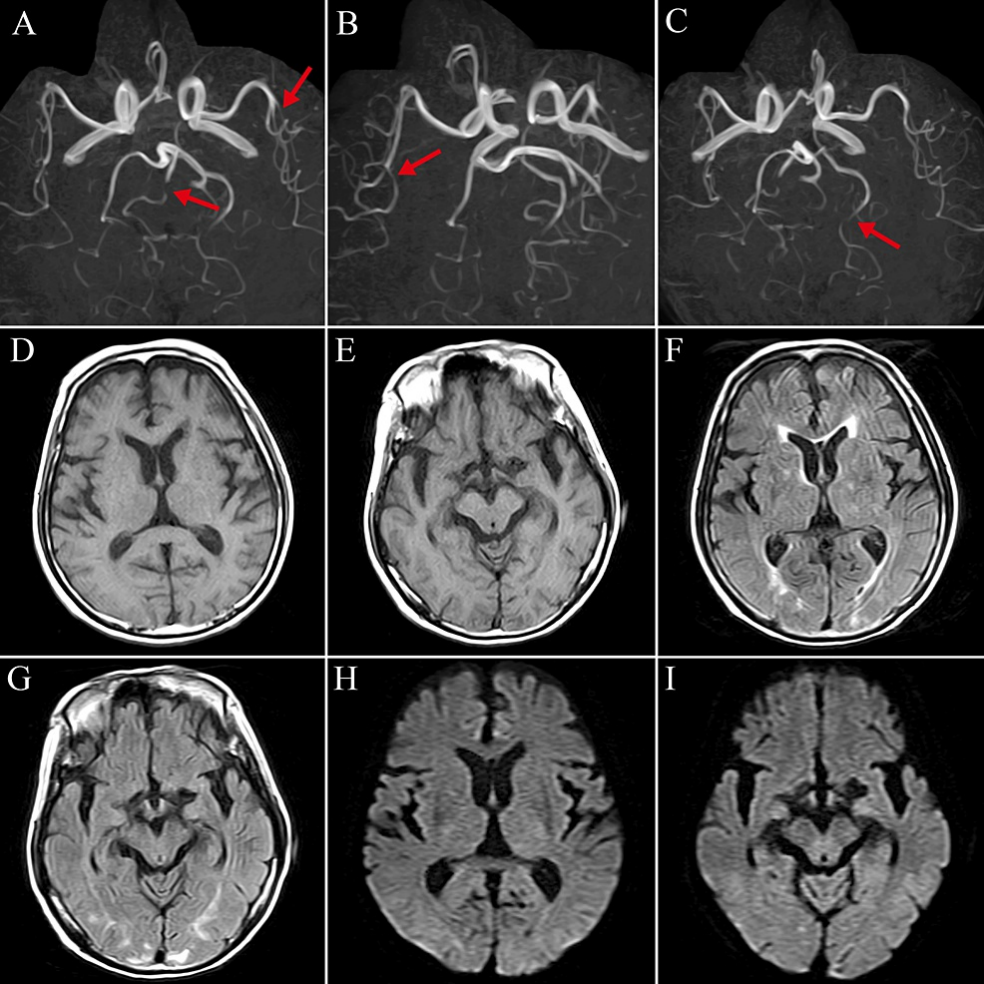
(A–C) Head magnetic resonance angiography showing multiple vasoconstrictions involving the middle and posterior cerebral arteries (red arrows). T1-weighted (D-E), FLAIR (F-G), and diffusion-weighted (H-I) images show chronic ischemic changes without any acute intracranial hemorrhage or infarction.

**Figure 2:**
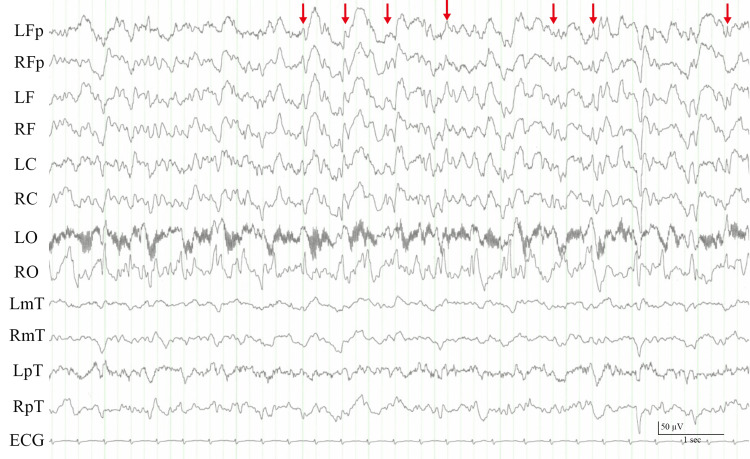
Electroencephalogram showing spike and wave discharges over the right hemisphere (red arrows). L: left; R: right; Fp: frontal pole; F: frontal; C: central; O: occipital; mT: mid-temporal; pT: postero-temporal.

On the 46th day after admission, the patient received a third SARS-CoV-2 vaccination (BNT162b2, Pfizer-BioNTech, New York). The following morning, he developed a moderate headache that was relieved with acetaminophen. There were no other symptoms at that time. A few hours later, the headache relapsed with tonic-clonic seizures and a disturbance of consciousness with a blood pressure of 195/128 mmHg. His Glasgow Coma Scale score was E4V4M5. On physical examination, there were no significant abnormalities. Neurological findings were unremarkable except for the conjugate deviation of the eyes to the right. Magnetic resonance imaging of the head revealed multiple vasoconstrictions involving the middle and posterior cerebral arteries, with no evidence of acute cerebral hemorrhage or infarction (Figure 1). Electroencephalography revealed frequent spike activity, dominant in the right hemisphere, and diffuse high-voltage slow waves (Figure 2). Intravenous diazepam (5 mg) stopped the tonic-clonic seizures. Although levetiracetam (1,000 mg/day) was initiated, intermittent convulsions of the left upper extremity and left side of the face persisted. Options for escalation of therapeutic intensity in the ICU, under intubation and ventilator management, were discussed among our team. However, since the patient had advanced COPD and the family did not want further treatment, no medication other than diazepam and levetiracetam was given after shared decision-making with the family. His cognitive and cardiorespiratory conditions gradually deteriorated, and he died 40 hours after vaccination.

**Figure 3:**
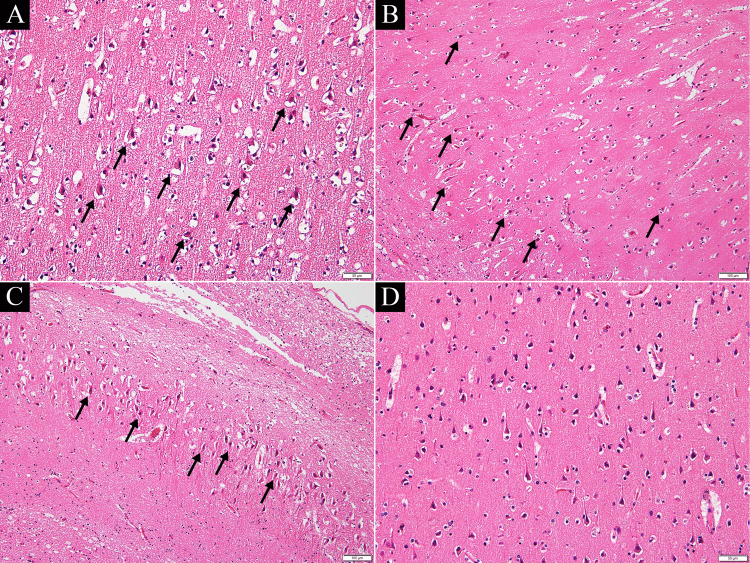
Pathological examinations at autopsy (hematoxylin and eosin stain). Severe perineuronal space enlargement and many eosinophilic neurons were observed in the temporal cortex (A), CA1 (B), and CA2–3 (C) (black arrows). Conversely, no pathological changes were observed in the anterior lobe (D).

The brain autopsy revealed multiple fresh ischemic lesions of various severities in the cerebral cortices of the temporal and occipital lobes, as well as in the Ammons horns (CA1-3) on both sides (Figure 3A-3C). No pathological changes were observed in the anterior lobe, basal ganglia, thalamus, brainstem, or cerebellum. There was no evidence of thrombus formation or inflammatory cell infiltration in the main cerebral arteries. From the above, it was inferred that extensive acute ischemic brain damage was caused by multiple vasospasms of the bilateral cortical branches of the middle and occipital cerebral arteries, that is, RCVS.

## Discussion

Here, we report a case in which the cause of sudden death one day after the SARS-CoV-2 vaccination was found to be RCVS on autopsy. In this case, the vaccination appeared to have resulted in RCVS. The findings in this case suggest the pathophysiology of sudden death and neurological side effects, including headache, which is one of the most common complications of the SARS-CoV-2 vaccination.

The adverse effects of the SARS-CoV-2 mRNA vaccine include pain at the injection site, fatigue, headache, and myalgia. Approximately 50-70% of patients experience headaches [1]. These headaches can be tension-type, intracerebral bleeding, subarachnoid hemorrhage, venous sinus thrombosis, or RCVS, which is less common [2].

RCVS is a clinical-radiological syndrome characterized by a thunderclap headache and reversible widespread vasoconstriction. Although it can affect both sexes and any age group, females in their forties are the most susceptible to RCVS. The pathophysiological mechanisms are not fully understood; however, they are assumed to include two main mechanisms: sympathetic overactivity and endothelial dysfunction. Patients with RCVS typically report triggers, including the use of vasoactive medications, postpartum status, and physical exertion [3,4]. Calabrese et al. established the diagnostic criteria for RCVS [5]. While the overall prognosis of RCVS is good and more than 90% of patients have good outcomes, some develop critical complications (e.g., convulsion, stroke, and subarachnoid hemorrhage) leading to death. Treatment includes the elimination of any precipitating or aggravating factors and the administration of calcium channel blockers (e.g., nimodipine) [3,4].

Recently, COVID-19 and its vaccines have been identified as possible causes of RCVS. Table 1 presents a review of the literature on RCVS following the COVID-19 vaccine. We conducted a search of the literature in the PubMed database published in English. The search was updated to April 21, 2024. The terms used were “reversible cerebral vasoconstriction syndrome," "COVID-19," "SARS-CoV2," and “vaccination.” To the best of our knowledge, this is the first autopsy case report of RCVS following a SARS-CoV-2 infection or vaccination. While the precise mechanism is not elucidated, the following mechanism is presumed [8]: angiotensin-converting enzyme 2 (ACE2) is assumed to play an important role. ACE2 is expressed in organs such as the heart, lungs, gastrointestinal tract, and blood vessels. It downregulates the vasoconstrictive peptide angiotensin 2 to the vasodilative peptide angiotensin 1-7. The SARS-CoV-2 spike protein interacts with ACE2 to promote cellular entry, leading to infection. As a result, ACE2 was downregulated and angiotensin-2 increased. This induces vasoconstriction and, possibly, RCVS. In contrast, mRNA vaccines are composed of stabilized mRNA that encodes spike proteins. It is assumed that after vaccination, the vaccine-expressed spike protein might similarly interact with ACE2, leading to vasoconstriction and RCVS.

**Table 1:** A review of literature of cases of reversible cerebral vasoconstriction syndrome after COVID-19 vaccine. d: days; F: female; h: hours; M: male; mRS: modified Rankin scale; NR: not reported.

Case no. [Ref]	1 [6]	2 [6]	3 [6]	4 [6]	5 [6]	6 [6]	7 [6]	8 [7]	9 [8]	Our case
Age	41	41	51	75	69	56	47	38	30	73
Sex	M	M	M	F	F	F	F	F	M	M
Symptoms	Dizziness, nausea, emesis, weakness, and speech impairment	Headache	Headache, nausea, and emesis	Headache	Headache, disorientation, and speech impairment	Headache	Headache	Headache, visual impairment	Headache	Headache
Time from COVID-19 vaccine to RCVS diagnosis	4 d	5 d	4 d	1 d	8 d	36 d	48 d	18 d	12 h	24 h
Outcome	NR	NR	NR	Unspecified improvement	NR	Improvement in headache	Improvement in headache	Mild visual impairment	No symptoms	Death
Autopsy	NR	NR	NR	NR	NR	NR	NR	NR	NR	Yes

Based on the autopsy findings, we concluded that the patient had an acute ischemic brain injury, mainly in the cortex of the middle and posterior cerebral artery territories, due to RCVS. Possible differential diagnoses were status epilepticus (caused by factors other than RCVS), hypoxic-ischemic encephalopathy, and systemic circulatory disturbances; however, none of these were considered unlikely. First, hippocampal injury due to status epilepticus usually occurs in the CA1, 3, and 4 regions, whereas CA2 tends to be preserved [9], which is not consistent with the pathological findings in the present case. In addition, there were no obvious lesions in the cerebellum or thalamus (dorsal medial nuclei), which are frequently affected by epilepsy. Second, areas easily affected by hypoxic-ischemic encephalopathies, such as the cerebellar, visual, motor, and sensory cortexes, were intact in this case [10,11]. Accordingly, the patient did not appear to have hypoxic-ischemic encephalopathy. Third, because there were almost no lesions in the watershed area, the possibility of death due to systemic circulatory disturbances was considered low [12]. Considering the above and the fact that it occurred the day after vaccination, RCVS triggered by SARS-CoV-2 mRNA vaccination could result in severe headache and status epilepticus, leading to extensive acute ischemic brain injury and death. Since the vasospasm of the vertebrobasilar artery was relatively mild, it is assumed that it did not lead to ischemic changes in the blood supply areas of the vertebrobasilar artery that could be seen in pathology.

## Conclusions

Headache is the most common adverse neurological effect of the SARS-CoV-2 vaccination. The causes of headaches vary and include RCVS. RCVS is generally a disease with a good prognosis but can have fatal complications such as seizures and strokes. If patients complain of headaches after the SARS-CoV2 vaccination, imaging studies, including MRI and MRA, should be considered. In this case, after the SARS-CoV-2 vaccination, RCVS was diagnosed both radiographically and pathologically. This can lead to a better understanding of the mechanism of headache as an adverse effect of the SARS-CoV-2 vaccination.
